# Noninvasive quantitative assessment of oral submucosal fibrosis *in vivo* using optical coherence elastography

**DOI:** 10.1117/1.JBO.30.12.124510

**Published:** 2025-10-17

**Authors:** Yuhao Yang, Chaoqun Ye, Mengzhen Tang, Zekun Li, Xinyu Yang, Xingdao He, Weihua Chen, Jian Yang

**Affiliations:** a Nanchang University, Jiangxi Medical College, School of Stomatology, Nanchang, China; b Jiangxi Province Key Laboratory of Oral Diseases, Nanchang, China; c Jiangxi Province Clinical Research Center for Oral Diseases, Nanchang, China; d Nanchang Hangkong University, Key Laboratory of Opto-Electronic Information Science and Technology of Jiangxi Province and Jiangxi Engineering Laboratory for Optoelectronics Testing Technology, Nanchang, China

**Keywords:** oral mucosal fibrosis, precancerous lesions, optical coherence elastography, elasticity imaging

## Abstract

**Significance:**

Optical coherence elastography (OCE) is a noninvasive imaging technique with high sensitivity and resolution that can be used for mucocutaneous imaging. Oral submucous fibrosis (OSF) is a chronic disease that has a tendency to become cancerous. Nevertheless, there are a few noninvasive methods for early detection of OSF.

**Aim:**

A piezoelectric transducer-based (PZT) OCE technique was devised to noninvasively assess the structural and mechanical properties of mucosa in healthy and fibrotic oral diseases.

**Approach:**

We first validated the accuracy and reliability of the OCE system for tissue elasticity detection by means of a heterogeneous agar model. The structural and biomechanical characteristics of the regional tissues were then evaluated by examining the oral mucosa of both healthy and fibrotic SD rats.

**Results:**

Normal and fibrotic tissue stiffness differed significantly (p<0.05). The elastic wave velocity was 6.44±0.30  m/s in the normal group and 14.2±0.91  m/s in the fibrotic group. After converting the results to Young’s modulus, the stiffness of the healthy buccal tissues and the fibrotic buccal tissues were 130.71±12.01 and 636.15±79.17  kPa, respectively (p<0.05).

**Conclusions:**

OCE can differentiate between normal and fibrotic tissue based on elasticity and optical properties. Healthy buccal tissues were softer than diseased tissues.

## 1 Introduction

Oral submucosal fibrosis (OSF), first reported in the mid-20th century, is a persistent, difficult to diagnose, and refractory condition with a tendency to become cancerous^1^^,^^2^ WHO has included it in the list of oral potentially malignant disorders.^3^^,^^4^ In total, 7% to 30% of patients with OSF may transform into oral squamous cell carcinoma (OSCC).^5^^,^^6^ OSCC transformed from OSF is characterized by higher aggressiveness, higher recurrence rates, and lower survival rates than OSCC transformed from non-OSF.^7^ Due to its propensity for malignancy, the management of OSF focuses on a combination of prevention and treatment.^8^^,^^9^ However, due to the complexity of the pathogenesis, there is still a lack of effective treatment.^9^ Early detection and diagnosis is the key to reducing the risk of malignant transformation of OSF and the risk of death.^10^ However, typical clinical symptoms of OSF are uncommon, which can easily lead to missed diagnosis and misdiagnosis.

Currently, the gold standard for diagnosing OSF is still histopathology, but biopsy testing is long and invasive, cannot be performed frequently, and is not suitable for early diagnosis.^11^ Therefore, we need simpler, noninvasive, and more accurate screening and diagnostic tools to help improve the efficiency of early detection of OSF. In recent years, some scholars have advocated the discovery of OSF biomarkers by liquid biopsy as an aid to diagnosis.^12^ However, as of now, the specificity of these biomarkers is limited. In addition, optical imaging techniques such as autofluorescence spectroscopy, Fourier transform infrared spectroscopy, and optical coherence tomography (OCT) have been used to aid in the diagnosis of OSF.^12^^–^^14^ In particular, OCT technology has the benefits of noninvasive, real-time, excellent resolution, and label-free tissue imaging, which shows great potential in the adjunctive diagnosis of oral diseases.^15^

It is commonly believed that OSF results from a combination of decreased collagen degradation and increased collagen formation, which causes collagen fibers to be deposited in the oral tissues.^5^^,^^8^ This results in different degrees of mouth opening restriction and decreases the oral mucosa’s elasticity. Therefore, the study of biomechanical properties of oral mucosa is important for the early diagnosis of oral mucosal fibrosis. Sample deformation, vibration, or shear wave propagation can be detected using optical coherence elastography (OCE), which combines elastography and OCT.^16^^–^^18^ Currently, the OCE technique has carried out a wide range of research applications in ophthalmology,^19^^–^^21^ breast,^22^^–^^24^ prostate,^25^ skin,^26^^,^^27^ and brain,^28^ providing new evidence for clinical studies. As a result, the method for evaluating biomechanical properties has been thoroughly validated.

In this paper, we describe the use of optical elastography to characterize oral mucosal tissues. We used a piezoelectric transducer-based (PZT) OCE system to evaluate the biomechanical characteristics of oral mucosa. Tissue deformation and elastic wave propagation were induced using a customized contact probe that combined a PZT and a homemade metal probe tip. Our results showed significant differences in the structure and elasticity of normal and diseased oral mucosa. This study confirms the unique advantages of OCE for early detection of subtle alterations in the structure and biomechanics of diseased tissue in oral mucosal tissues.

## 2 Materials and Methods

### 2.1 System Setup

**Fig. 1:**
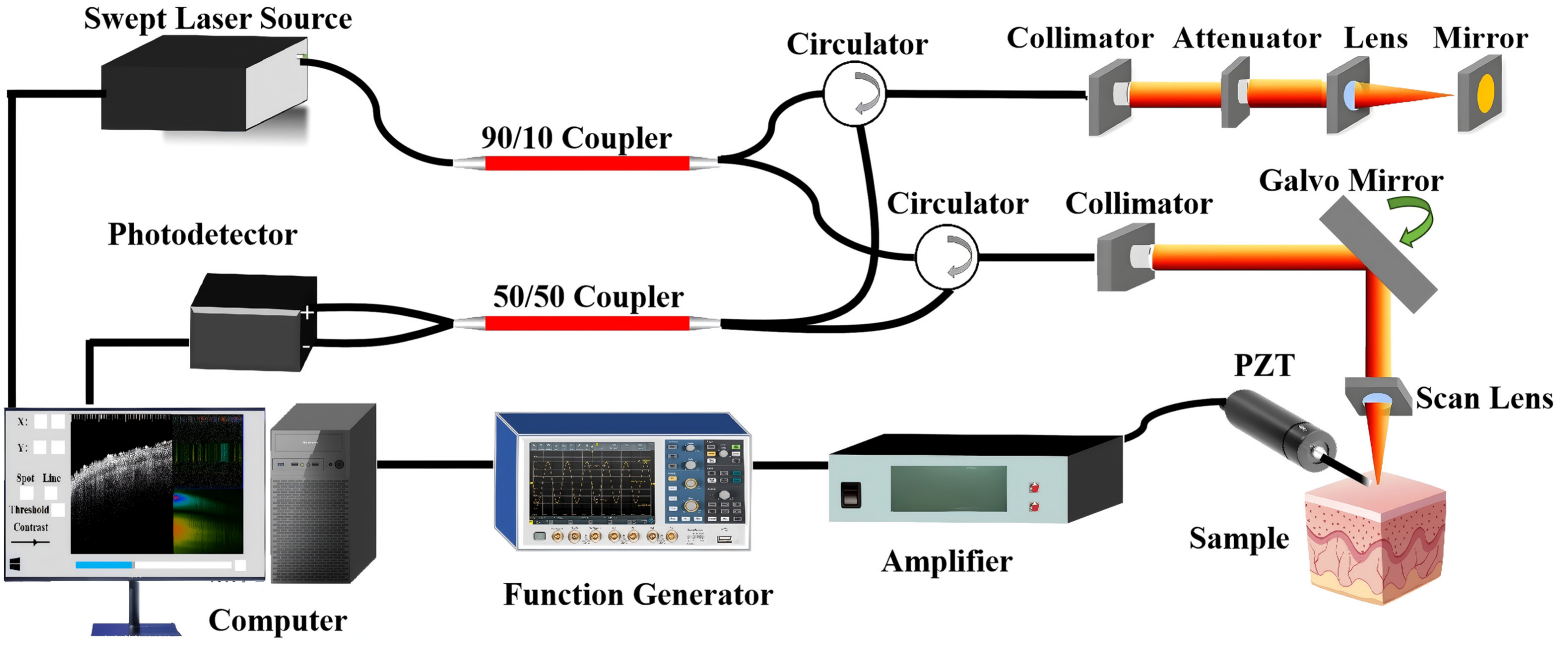
Schematic of the PZT-OCE system.

Figure 1 depicts the experimental setup schematically. Our study was conducted using a customized swept source optical coherence tomography (SS-OCT) system. It operates a scanning laser with a center wavelength of 1310 nm, a bandwidth of 110 nm, an A-line rate of 50 kHz, and an average output power of 20 mW. A 90:10 fiber optic coupler is used to split the laser’s light, with 90% of the output going to the sample arm and 10% to the reference arm. Following their reflection back from the sample and reference arms, the interfering signals pass through a 50:50 optocoupler before being captured by a balanced photodetector (PDB480C-AC, Thorlabs). An acquisition card (Alazar Technologies Inc., Quebec, Canada) for a waveform digitizer was used to digitize the detector signals. In order to efficiently control the direction of the optical path and improve the signal detection efficiency, we added a circulator to the system for separating incident and reflected light to avoid the reflected light from interfering with the stability of the light source.

In order to excite the tissue, the excitation section employed a specially designed contact probe that paired a custom-made metal probe tip with an integrated PZT. The cross-sectional area of the probe tip was approximately 0.4 mm in diameter. The PZT drive signal is driven at 1 KHz, with a peak-to-peak voltage of 3.3 V as the amplifier’s start-up voltage, and a contact force on the order of microNiu (μN) to avoid tissue damage. The function generator produces a continuous pulse signal causing the PZT probe to vibrate periodically, and this external excitation acts on the biological tissue, inducing a continuous elastic wave inside the tissue, which is subsequently captured by the OCT system. During the measurement process, the signal processing software is used to correct the measurement signal using a signal compensation algorithm, and adaptive filtering technology is used to effectively remove motion noise, improve the quality of the measurement signal, and effectively reduce the effect of any possible object movement.

### 2.2 Data Acquisition

**Fig. 2:**
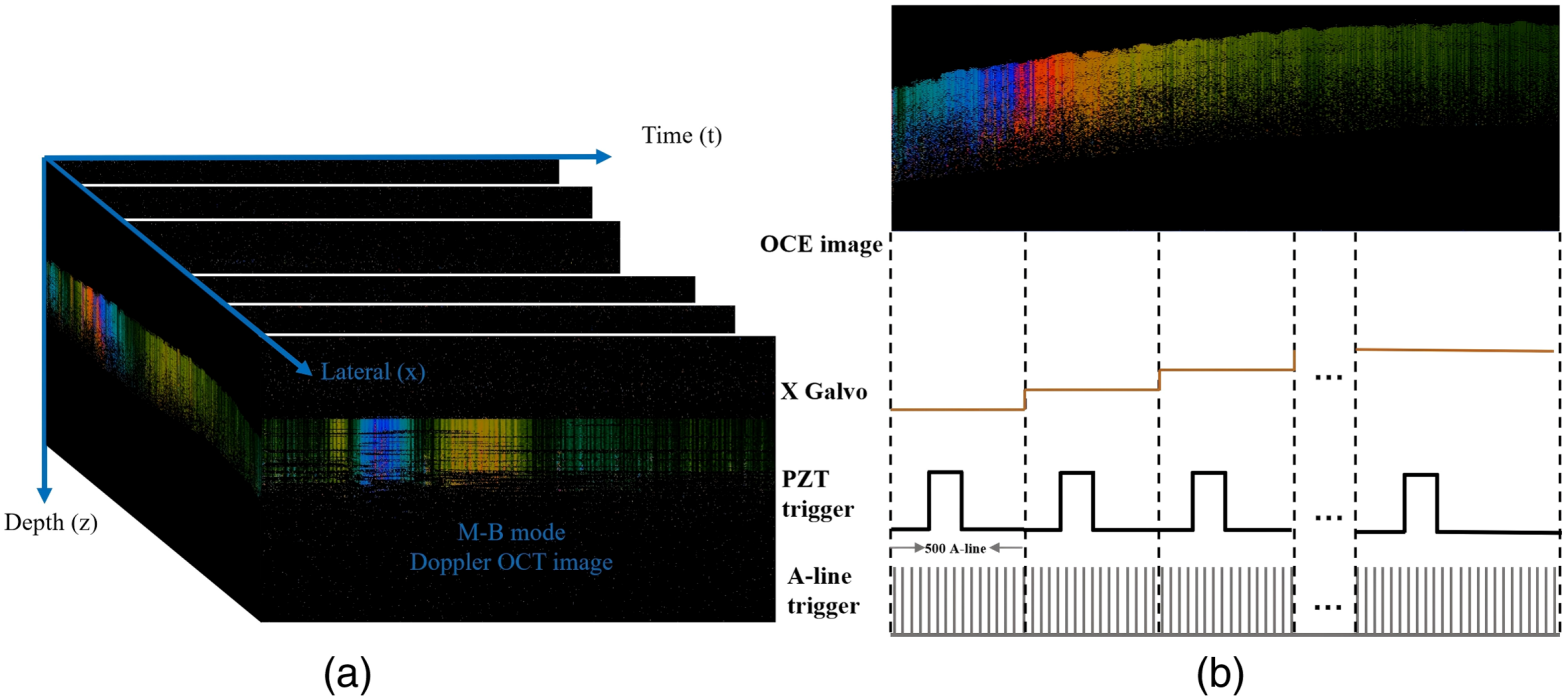
(a) Scan protocol of the M-B mode. (b) Timing diagram of the M-B mode.

The system was obtained using the MB protocol. The timing diagram of the MB modes is shown in Fig. 2. During elastic imaging of biological tissues, one cycle scan was performed under the same position, and a total of 500 A-lines were scanned in this cycle. Between the 101st A-line and 120th A-line, the excitation source received a trigger signal with a duration of 0.4 ms, and at this time, the scanning galvanometer was kept motionless in the X-axis direction, and one M-scan image was completed. The scanning beam was then moved to the next position, and the same scan was repeated until a total of 1000 positions in the lateral direction were completed, and the time-delayed Doppler B-scan image was reconstructed by M-scan.

### 2.3 Experimental Program

#### 2.3.1 Phantom generation and measurement

First, we verified the reliability and trustworthiness of the OCE system for tissue elasticity detection by using a heterogeneous agar model. Heterogeneous agar models with concentrations of 0.7% and 0.5% were prepared according to reference Zhu et al..^29^ Structural images and elastic wave velocity were obtained, and Young’s modulus values were computed by repeating the experiment 3 times.

#### 2.3.2 Sample preparation

This study’s animal experiments were authorized by the Animal Care and Use Committee of Nanchang University, Ethics No. NCULAE-20240827001. Twelve SD male rats, aged 5 weeks and weighing about 200 g, were obtained from GemPharmatech Co., Ltd (SCXK(SU) 2023-0009). The experimental animals were housed at room temperature of 18°C to 25°C, relative humidity of 30% to 50%, indoor ventilation, 24-h light-dark cycle conditions, and fed standard rat food. A total of twelve SD rats were split into six-rat experimental and control groups at random. Rats in the experimental group were injected with 0.1 ml of bleomycin hydrochloride (BLM) solution at a concentration of 1.0  mg/ml into the buccal mucosa bilaterally, while rats in the control group were injected with an equal volume of 0.9% saline, and the frequency of injection was 1 injection/3d, with fasting and water withdrawal for 2 h after the injection, and the injections were given for 16 weeks consecutively. The buccal mucosa of the rats was observed during the modeling period, and the body weights of the rats were recorded weekly. Under isoflurane anesthesia to moderate anesthesia, the distance between the maxillary and mandibular incisal segments was measured by vernier calipers as the degree of passive mouth opening (the opening tension was 2N). The average value, which was precise to 0.01 mm, was calculated by repeating the measurement 3 times.

#### 2.3.3 Animal measurement

OCE was performed on rats at week 16 of modeling. Prior to imaging, SD rats were anesthetized by intraperitoneal injection. The cheek pouches of the anesthetized animals were inverted and placed under the imaging system. Throughout the experiment, the cheek pouches were kept dry, and their elasticity was assessed using shear wave patterns. Each test should take no more than half an hour to obtain good results and ensure anesthesia. OCE was collected from the inspection pouch in three separate places. Tissue ink was used to mark these locations so that imaging and biopsy sites could be correlated. Three repetitions of each measurement were made to guarantee the accuracy and consistency of the data.

### 2.4 Histological Assessment

After OCE evaluation, rats were euthanized, and buccal mucosal tissues were excised for immediate histopathological processing. 10% formalin fixation for 24 h, paraffin embedding, routine tissue sectioning, and staining with hematoxylin and eosin (hematoxylineosin, HE) for histopathological observation. The tests remained impartial as the experimenter had no knowledge of the treatment of the animals during the experiment and the reviewer had no knowledge of the group assignment during the histologic analysis.

### 2.5 Statistical Analysis

The experimental data information was taken as mean ± standard deviation, using SPSS26.0 software statistical analysis, two independent samples t-test between the control group and the experimental group to analyze, p<0.05 indicates that the difference is statistically significant.

## 3 Result

### 3.1 Animal Histology Results

**Fig. 3:**
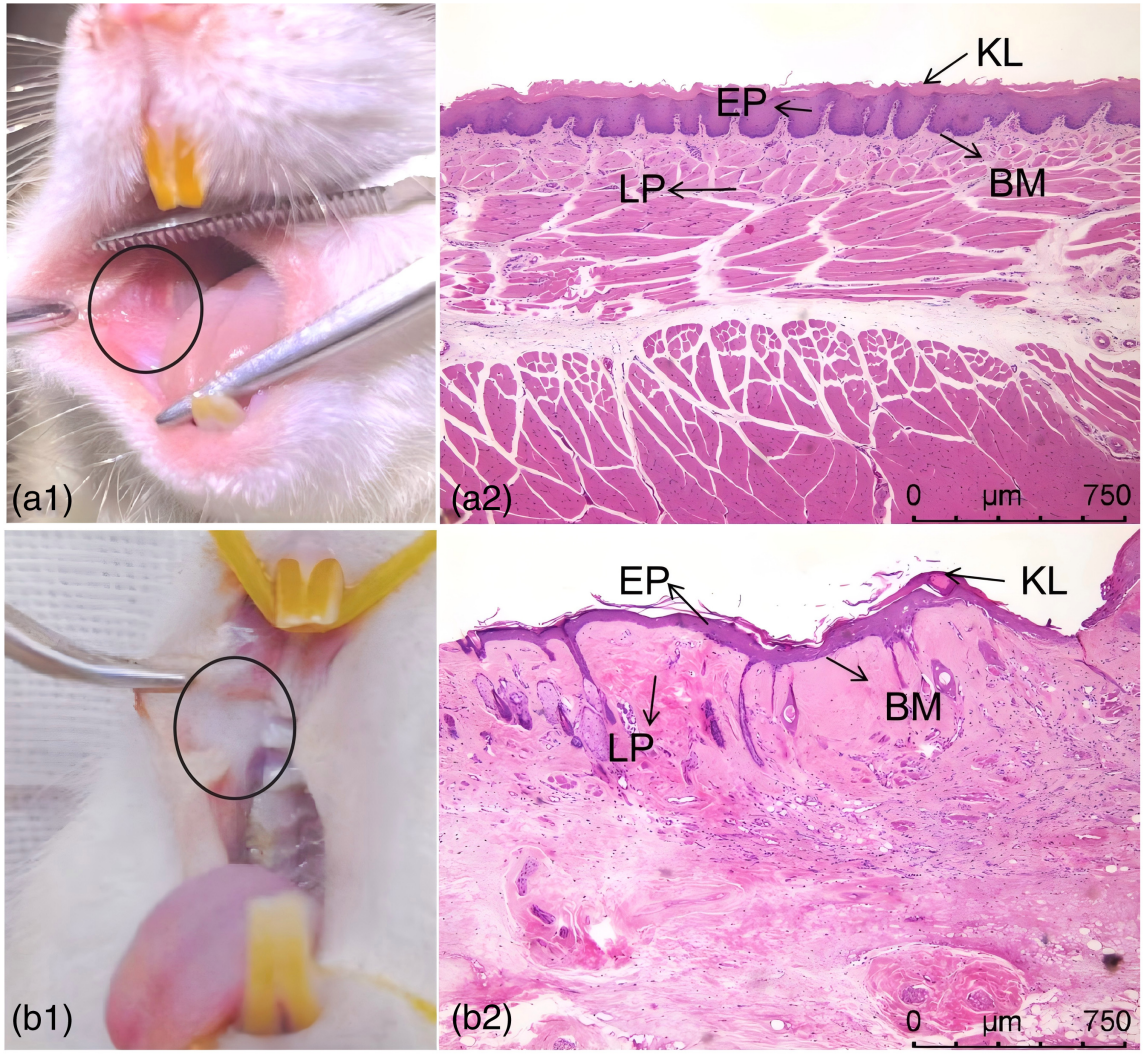
Photograph (a1) and HE staining image (a2) of healthy rat buccal mucosa. Photograph (b1) and HE staining image (b2) of fibrotic rat buccal mucosa. KL, keratin layer; EP, epithelial layer; LP: lamina propria; BM, basement membrane.

During the establishment of the OSF model, the color and texture of the oral mucosa of the rats in the experimental group had different degrees of changes, while the changes in the control group were not obvious [see Figs. 3(a1) and 3(b1)].The changes in the degree of opening of the mouth of the rats during the modeling of the OSF are shown in Table 1, and the differences in the degree of opening of the mouth of the rats in the two groups were statistically significant (p<0.05). The corresponding histologic results are shown in Figs. 3(a2) and 3(b2). In the experimental group, the epithelium of the buccal mucosa atrophied significantly, the epithelial spikes became flat or even disappeared, the lamina propria was not infiltrated by inflammatory cells, the collagen fibers in the lamina propria and the submucosa could be seen to be deposited, and some of them were vitreous, and the number of blood vessels was occluded and reduced compared with that in the control group [Fig. 3(a2)]. In the control group, the buccal mucosa was structurally normal, with no obvious hyperplasia or atrophy of the epithelium, visible nail protrusions, no inflammatory cell infiltration in the lamina propria, visible blood vessels, and no deposition of collagen fibers [Fig. 3(b2)].

**Table 1:** Changes in mouth opening in rats during OSFM modeling (mm).

	Pre-experimental	16th week
Experimental group	15.64 ± 1.43	6.89 ± 2.04
Control group	15.46 ± 1.84	21.23 ± 1.89

### 3.2 OCE Results

#### 3.2.1 Heterogeneous phantom imaging

**Fig. 4:**
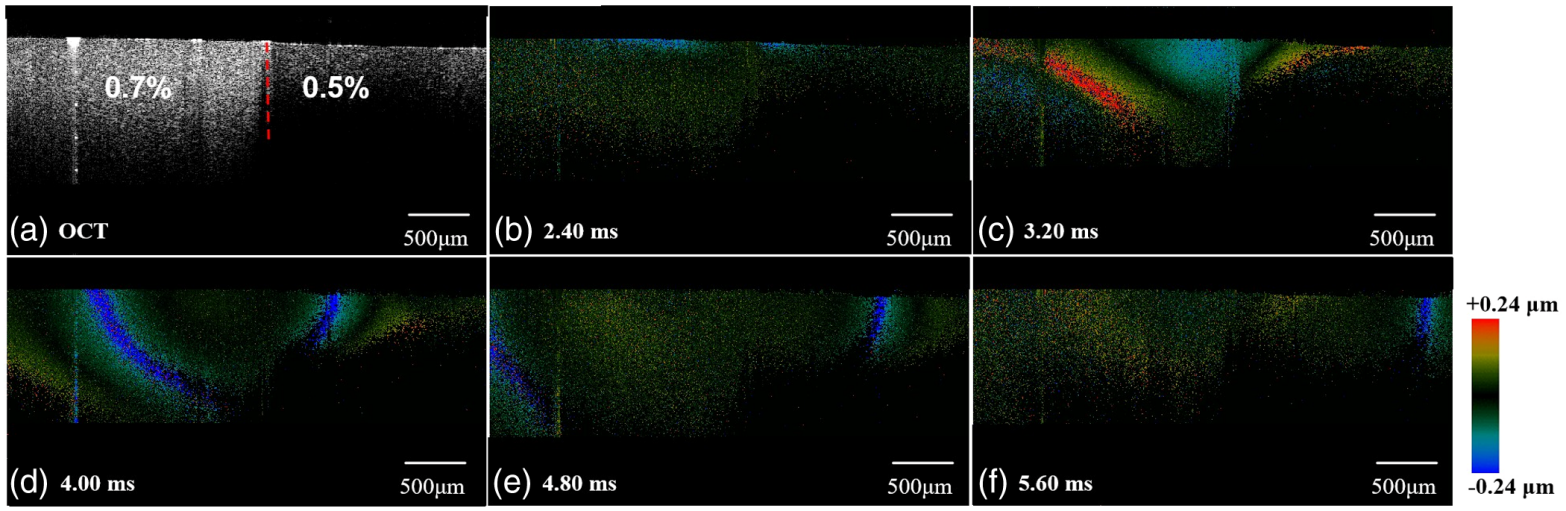
Imaging results of the heterogeneous phantom. (a) 2D OCT image, (b)–(f) Maps of shear wave propagation at different time points.

In order to verify the feasibility of the OCE system, experiments were first performed on the heterogeneous agar model. Figure 4(a) shows the B-scan image of the OCT structure of the heterogeneous body model. Small changes in agar concentration can have a significant effect on its internal microstructure. Higher concentration agar has a more uniform and dense molecular arrangement, which creates more scattering centers, increases the intensity of light received in the direction of detection, and reduces light loss due to structural inhomogeneities, which combine to make higher concentration agar samples brighter in the image. As a result, the left portion (0.7%) is brighter than the right portion (0.5%), and the intersection of the two agar concentrations is well defined and marked by a red dashed line. Figures 4(b)–4(f) show the elastic wave propagation images of heterogeneous agar at different time points, where the elastic wave propagates from the excitation position, i.e., the junction of the two concentrations of agar, to both sides.

**Fig. 5:**
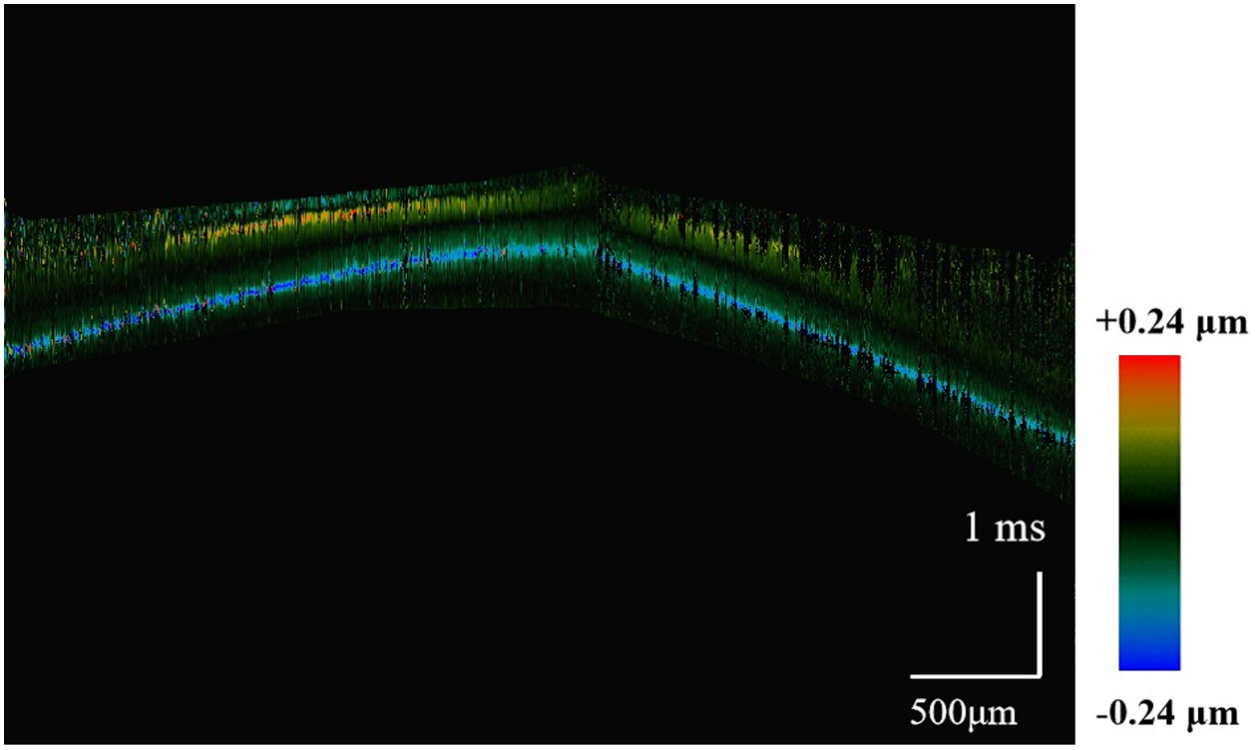
Spatiotemporal OCT image of shear wave of the phantom.

Figure 5 shows the spatio-temporal Doppler OCT images of the elastic wave propagation. It can be found that the slope of the left image is obviously smaller and the speed is larger compared to the right side, which is consistent with the conclusion that the speed increases with the larger agar concentration in the homogenized agar experiment. In order to reduce the measurement error, we measured the elastic wave velocity on each side of the agar 3 times, and the final results, the propagation velocity of the elastic wave in 0.7% and 0.5% agar on both sides of the heterogeneous agar were 2.87±0.09 and 1.67±0.05  m/s, respectively. From the Rayleigh wave model, we can get that the Young’s modulus of the heterogeneous agar on both sides of the 0.7% and 0.5% agar were 24.67±1.69  kPa and 8.35±0.49  kPa, respectively, which is similar to the values reported by CHEN’s group in the existing literature and is in line with the experimental expected results,^30^ which demonstrates the ability of the system to distinguish between tissues with different hardness.

### 3.3 *In Vivo* Rat Imaging

**Fig. 6:**
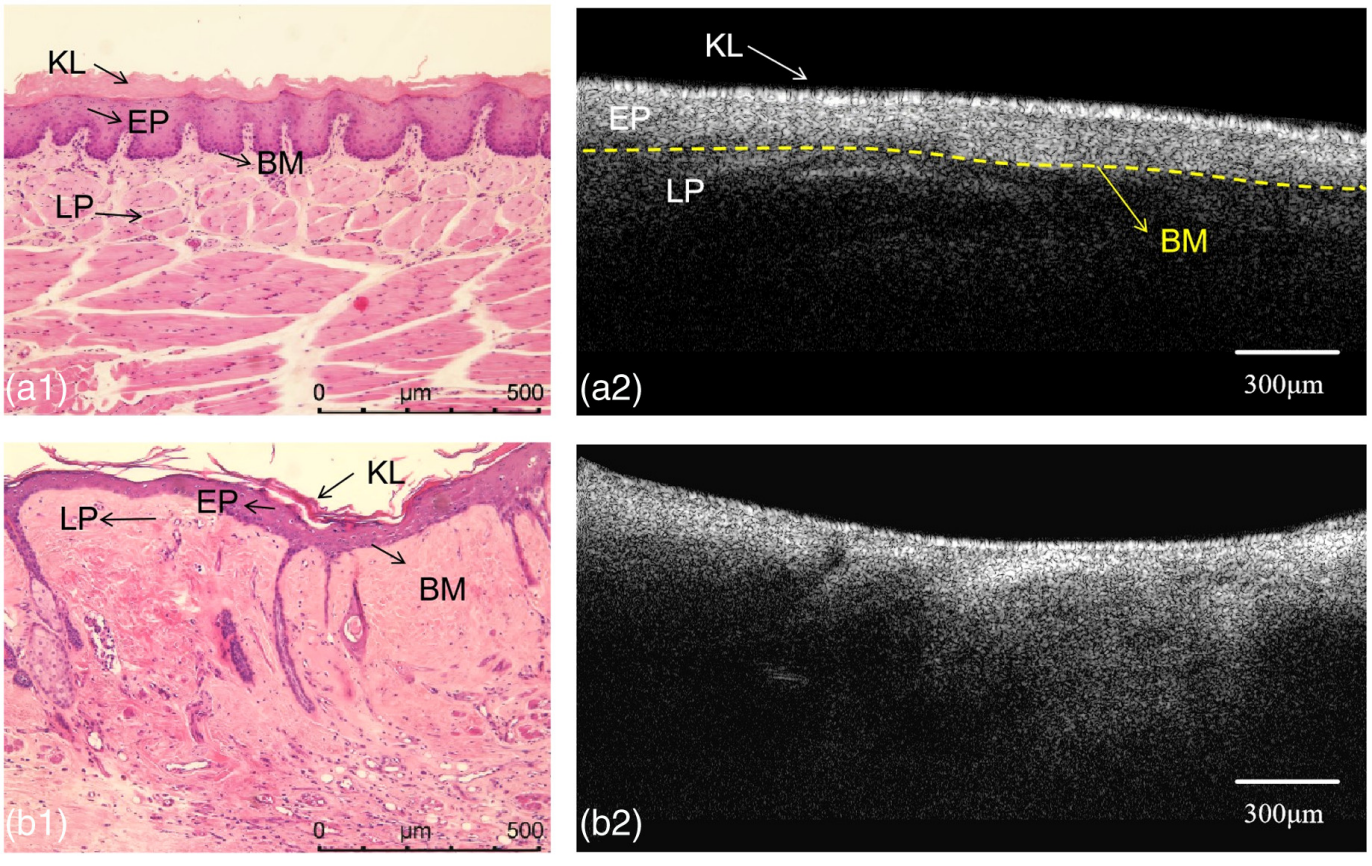
(a1) H&E-stained sections (a1) and *in vivo* OCT image (a2) of healthy rat buccal mucosa. H&E-stained sections (b1) and *in vivo* OCT image of fibrotic rat buccal mucosa. The yellow lines indicate the BM, which is the boundary between EP and LP. KL, keratin layer; EP, epithelial layer; LP: lamina propria; BM, basement membrane.

After systematic validation, OCE imaging of the buccal vesicles of live SD rats was performed using the same method to investigate the differences in viscoelastic properties between normal buccal vesicles and fibrotic tongues. Figures 6(a2) and 6(b2) show the 2D OCT structures of healthy buccal pouches in the control group and fibrotic buccal pouches in the experimental group, respectively. As shown in Fig. 6(a2), the OCT images of the healthy buccal mucosa showed a clear outline of the mucosal layers observed in matched histology, with the keratinized layer (KL), epithelial layer (EP), lamina propria (LP), and the basement membrane (BM) clearly visible, and the basement membrane was continuous and intact. In contrast, the B-scan of the fibrotic buccal pouch, as shown in Fig. 6(b2), showed poorly defined stratification and even some areas disappeared. The B-scan of the fibrotic tissue shows that the intensity is brightest at the surface and gradually decreases with increasing depth.

**Fig. 7:**
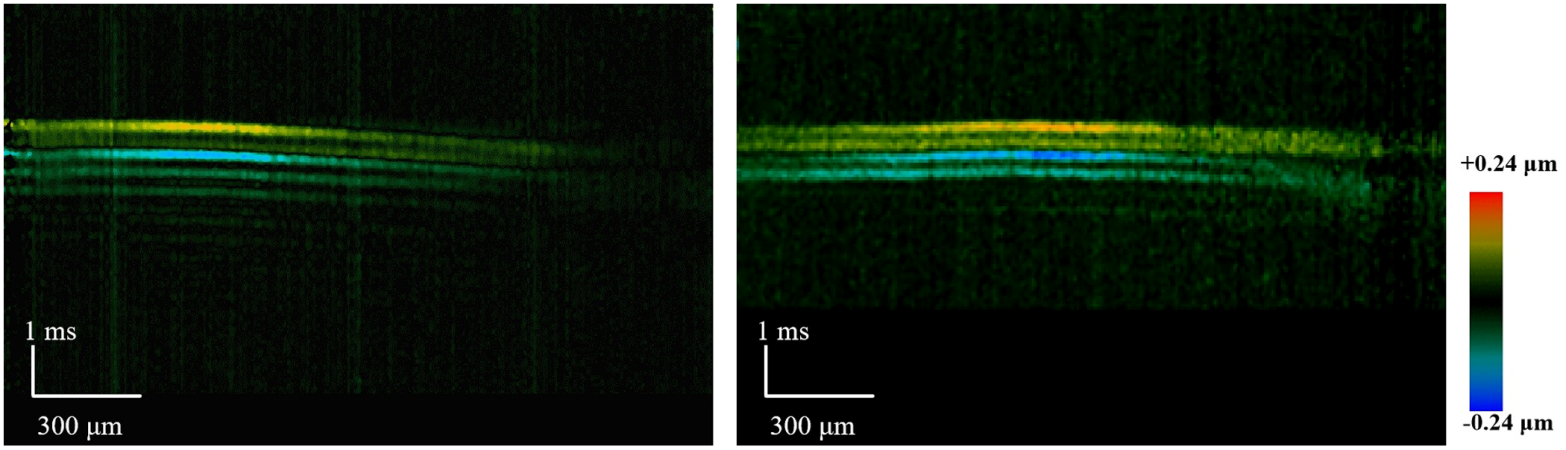
Spatiotemporal images of Rayleigh wave propagation. (Left:) healthy buccal mucosa. (Right:) fibrotic buccal mucosa.

In order to compare the elasticity differences, spatiotemporal Doppler images corresponding to the respective, as shown in Fig. 7; it can be observed that there is a significant difference in the slopes, which reflects the elasticity differences between normal buccal pouch tissues and fibrotic tissues. The stiffness of the normal buccal pouch was relatively low compared to the fibrotic buccal pouch.

**Fig. 8:**
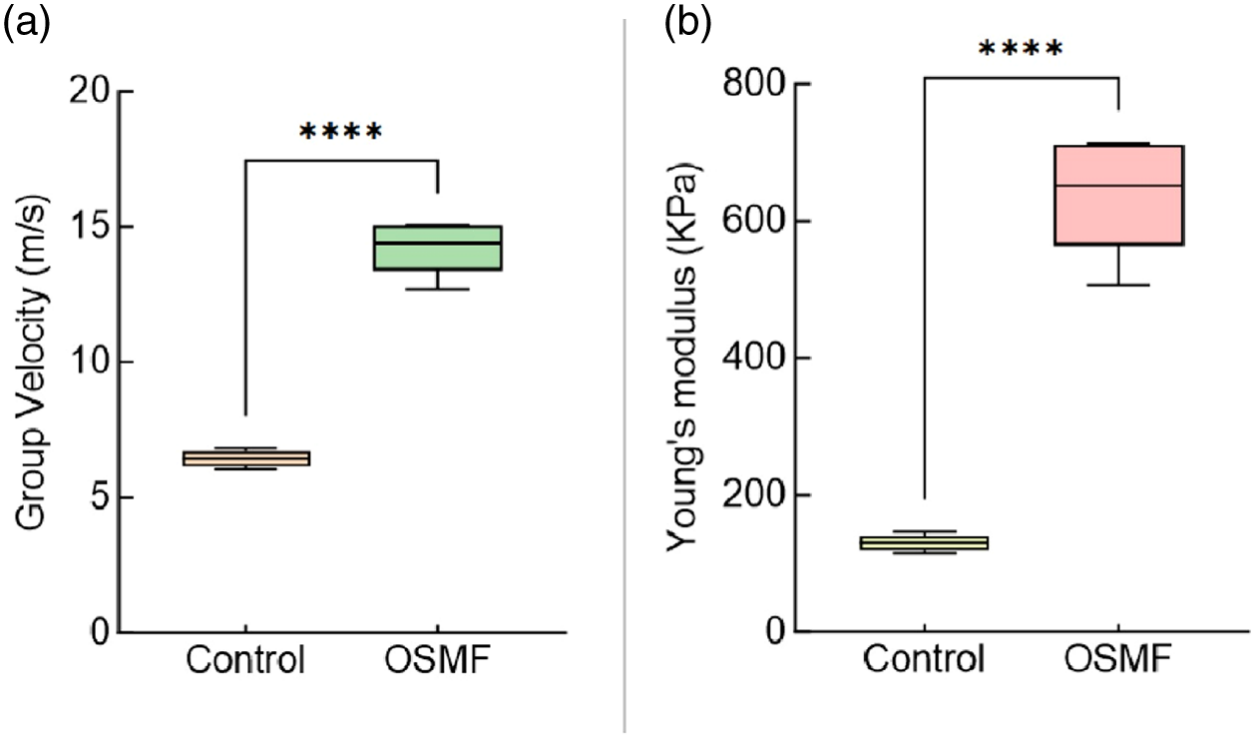
Biomechanical properties of healthy and fibrotic buccal mucosa. (a) Averaged phase velocity and (b) average Young’s modulus.

Figure 8 shows box-and-line plots of elastic wave velocity and Young’s modulus through the control and diseased samples. The boxes correspond to the interquartile range, and the inset box is the median. With the error being the standard deviation between samples, the control sample’s elastic wave velocity was 6.44±0.30  m/s, and the diseased sample’s was 14.2±0.91  m/s. A significant difference between the two groups was found using a two-sample t-test with unequal variances (p<0.05). After converting the results to Young’s modulus, the stiffness of healthy and fibrotic buccal pouches was 130.71±12.01 and 636.15±79.17  kPa, respectively (p<0.05).

## 4 Discussion

Oral mucosal diseases are characterized by a variety of subtypes and clinical manifestations, making early detection and diagnosis difficult.^12^ OSF is usually diagnosed in combination with clinical examination and biopsy.^12^ In the current study, the quantitative diagnostic method for OSF is the measurement of maximum mouth opening, i.e., from the incisal edges of the upper mesial incisors to the incisal edges of the corresponding lower mesial incisors.^31^ However, this method does not provide the true condition of the patient’s oral mucosa. In the present study, we demonstrate the first use of the OCE technique to detect structural and elastic differences between BLM-induced OSF produced by OSF and oral mucosa samples from healthy rats. Previous work reported that the BLM-induced OSF model was significantly similar to the naturally occurring OSF model in patients, which supports the clinical value of the results presented in this study.^32^

In order to identify biomechanical alterations in specific biological tissues, the PZT-OCE suggested in this work offered a higher resolution than traditional mechanical testing, ultrasound, and magnetic resonance imaging. Specifically, PZT-OCE is based on OCT technology, which has an OCT lateral resolution of up to 1 to 10  μm and an axial resolution of up to 2 to 15  μm, which is significantly better than ultrasound imaging (0.1 to 1 mm) and magnetic resonance imaging (1 to 3 mm), which have a resolution of millimeters.^18^ Compared with magnetic resonance elastography (MRE) and ultrasound elastography (UE), OCE’s high-frequency waves not only capture tissue microstructural deformation through micron-level spatial resolution, but also enable highly accurate measurement of shear modulus through microstrain-level ($10^{-6}$) sensitivity.^18^^,^^33^^,^^34^

We first verified the feasibility of the OCE system for tissue elasticity detection by using a heterogeneous agar model, which showed that the system was able to distinguish between tissues of different stiffness. Subsequently, we tested healthy and fibrotic tissues and obtained the corresponding structural and elastic information. Structural analysis showed that changes in the epithelium, the subepithelium, and changes in the borders of the basement membrane can be used as effective diagnostic indicators of lesions, reflecting the differences in tissue microstructure between control and fibrotic tissues. However, when early lesions with poorly characterized surfaces coexist with OSF in the patient’s mouth, early structural changes may make it difficult to distinguish between healthy tissue, lesions, and OSF.^12^^,^^35^ And the elasticity information is a complement to the structure. Young’s modulus was quantified as 130.71±12.01  kPa for healthy tissue and 636.15±79.17  kPa for fibrotic. The results of Young’s modulus for healthy and fibrotic were then summarized in a box line plot. The difference between them was clear, with fibrosis being significantly stiffer than healthy mucosal tissue. Closely related to previous work using ultrasound elastography.^36^^–^^38^ This suggests the potential for OCE to differentiate between healthy and fibrotic buccal pouch tissue based on what can be structural and elastic indicators.

In this study, we detected BLM-induced OSF in rat oral mucosal tissues by combining an optical and biomechanical evaluation. However, the results showed that this optical imaging method was able to successfully differentiate between fibrotic and healthy mucosal tissues. Nonetheless, the high scattering of oral mucosal tissues could lead to bias in the optical property analysis.^39^^,^^40^ Because OCE cannot image the entire sample, it is limited in its ability to analyze the visible depth of mucosal tissue.^27^ Sample thickness, curvature, and boundary conditions during OCE measurements can also affect the elasticity of the measurements.^41^^,^^42^ Additionally, to guarantee that the patterns observed here are reliable and repeatable, a larger sample size than the one used in this work is required. In conclusion, this is a preliminary study to suggest the potential use of OCE technology in the diagnosis of healthy and fibrotic tissues in the oral cavity. Future research should focus on developing motivational strategies and quantitative analysis methods to accurately measure the viscoelasticity of oral mucosal tissues in order to provide potential evidence for the early diagnosis of OSF.

## 5 Conclusion

We used the OCE technique for the first time to identify elastic changes in oral mucosal tissues affected by BLM-induced OSF, and the OCE and histopathologic results were in excellent agreement, demonstrating the feasibility of OCE to detect mucosal changes associated with OSF. According to preliminary estimates, diseased tissue was stiffer than healthy tissue. In addition, structural analysis of the tissues showed differences in the optical structure of the tissues between healthy tissues and tissues with OSF. In conclusion, OCE is a promising technique for the diagnosis and monitoring of OSF.

## 6 Appendix: Data Processing

The phase-resolved Doppler algorithm was used to determine the phase difference Δφ between two adjacent A-lines in the continuous data acquisition time using the M-mode OCT images, as indicated in Eq. (1):^43^

Δφ=tan−1Im(Fm×Fm+1*)Re(Fm×Fm+1*). (1)

The complex data for a position and its subsequent position were $F_{m}$ and $F_{m+1}$, respectively. The conjugate of $F_{m}$ was represented by $F{*}_{m+1}.I_{m}()$ and $R_{e}()$ represented the imaginary and real components of the original data, respectively. The axial displacement change Δd can be calculated by Eq. (2):^44^

$$\Delta d=\frac{\lambda_{0}}{4\pi n}\Delta \varphi .$$ (2)

The laser’s central wavelength was represented by $\lambda_{0}$, and its refractive index was indicated by n. The displacement difference can be used to create a Doppler OCT image, which can then be used to extract data about the temporal and spatial propagation of shear waves. Equation (3) was utilized to determine the shear wave velocity Vs based on the displacement of the shear wave over time:^45^

$$Vs=\frac{\Delta x}{\Delta t}.$$ (3)

The displacement and propagation time of shear waves were Δx and Δt, respectively. Equation (4) showed the relationship between shear modulus and shear wave speed:^46^

$$\mu =\rho \times V_{S}^{2}.$$ (4)

ρ, V s and μ refer to the density of the buccal (estimated to be $1000\;\mathrm{kg}/m^{3}$^47^), shear wave velocity and shear modulus, respectively. Equation (5) can be used to calculate the Young’s modulus of buccal tissue based on the relationship between shear modulus and Young’s modulus:^48^

E=2μ(1+ν). (5)

Poisson’s ratio is assumed to be v=0.5.^49^ Equation (6) further can be simplified for the Young’s modulus:

$$E=3\times \rho \times V_{s}^{2}.$$ (6)

## Data Availability

The data and the associated code that support the findings of this study are available from the corresponding author upon reasonable request and through a collaborative research agreement.
